# Factors associated with maintenance of antibody responses to influenza vaccine in older, community-dwelling adults

**DOI:** 10.1186/s12879-015-0926-8

**Published:** 2015-04-23

**Authors:** H Keipp Talbot, Laura A Coleman, Yuwei Zhu, Sarah Spencer, Mark Thompson, Po-Yung Cheng, Maria E Sundaram, Edward A Belongia, Marie R Griffin

**Affiliations:** Vanderbilt University Medical Center, A2200 MCN 1161 21st Ave, Nashville, TN 37232 USA; Abbott Nutrition, 3300 Stelzer Avenue, Columbus, OH 43219 USA; Centers for Disease Control and Prevention, 1600 Clifton Rd MS A32, Atlanta, GA 30333 USA; University of Minnesota, Minneapolis, MN 55455 USA; Marshfield Clinic Research Foundation, 1000 N. Oak Avenue, Marshfield, WI 54449 USA

**Keywords:** Influenza, Older adults, Immunogenicity, Vaccine

## Abstract

**Background:**

Little is known about factors associated with maintenance of hemagglutinin inhibition (HAI) antibodies after influenza vaccination in older adults.

**Methods:**

Adults ≥50 years of age were vaccinated prior to the 2009–10 influenza season. Serum was drawn pre-vaccination (S1), 21–28 days post-vaccination (S2), and after the influenza season (S3) for HAI assays. Seroconversion was defined as ≥ 4-fold increase S1 to S2 (or if S1 < 10, by an S2 ≥ 40) and seroprotection was defined as S2 ≥ 40. Maintenance of antibody response was measured in participants with an S2 ≥ 40, and defined as an S3 ≥ 40.

**Results:**

We enrolled 510 participants during Fall 2009 at Vanderbilt University Medical Center and Marshfield Clinic Research Foundation. Participants’ mean age was 64 years with 62% female and 96% white. Seroconversion and seroprotection rates were lowest for influenza A H1N1 (12% and 26%, respectively), highest for influenza A H3N2 (45% and 82%), and intermediate for influenza B (28% and 72%). Of the participants with an S2 ≥ 40, 36% (46/126), 71% (289/407), and 74% (263/354) maintained an S3 ≥ 40 for H1N1, H3N2, and B influenza vaccine strains, respectively. S1 HAI titer was strongly associated with both post-vaccination seroprotection and maintaining seroprotection at S3 for all three influenza antigens. Age, sex, body mass index, self-reported stress, and vaccination site were not consistently associated with vaccine response or maintenance of response.

**Conclusions:**

Pre-vaccination antibody titer was the only study variable consistently and positively associated with both serologic response to vaccination and maintenance of response. Antibody responses were lowest for the H1N1 vaccine strain.

**ClinicalTrials:**

gov Identifier: NCT02401893

**Electronic supplementary material:**

The online version of this article (doi:10.1186/s12879-015-0926-8) contains supplementary material, which is available to authorized users.

## Background

In the United States, yearly influenza vaccination begins in August or September. However, influenza season can extend into April of the following year. The duration of protection from annual vaccination in older adults is unknown, and little is known about factors associated with the maintenance of response throughout the influenza season. This study evaluated factors associated with the immune response of older adults to trivalent influenza vaccine and the maintenance of antibody responses for the duration of the influenza season.

## Methods

### Subjects

Subjects were enrolled at two sites, Vanderbilt University Medical Center (Nashville, TN) and Marshfield Clinic Research Foundation (Marshfield, WI), during September and October 2009. Subjects were eligible for recruitment if they were ≥50 years of age and had no contraindication to influenza vaccination. Subject recruitment included advertisements at Vanderbilt University Medical Center and letters of invitation to older adults who had received an influenza vaccine in the year prior at Marshfield Clinic [1]. All subjects were vaccinated either by their usual caregiver or by the study staff. Strain components for the 2009–2010 Northern Hemisphere vaccine included A/Brisbane/59/2007-like (H1N1), A/Brisbane/10/2007-like (H3N2), and B/Brisbane/60/2008-like. Participants were given the trivalent seasonal vaccine because the 2009 H1N1 pandemic vaccine was not available.

### Data collection

All subjects donated serum pre-vaccination (S1) during September through October 2009, 21–28 days post-vaccination (S2) and post-influenza season, May through July, 2010, approximately 250 days (8 months) post-vaccination (S3). We chose the post-influenza season blood draw to be 8 months following S1 since this is likely to be the maximal duration of needed protection for a given influenza season. In the US, some influenza vaccines are now being given in August, and it is not unusual for the influenza season to extend into March and April. Study procedures, informed consent documents and data collection forms were reviewed and approved by Institutional Review Boards at each of the study sites.

Age, co-morbid conditions, sex, and race were ascertained from participant interview. Recent chemotherapy, radiation therapy, or use of immunomodulating medications were ascertained by self-report or chart review. CDC-defined high risk medical conditions were identified by self-report of organ transplantation, cancer, diabetes mellitus, splenectomy (functional or anatomic), cardiovascular disease, renal disease, sickle cell disease, chronic pulmonary disease, seizure disorder, immune deficiency, or dementia [2]. Self-reported stress was determined by asking participants to respond yes or no to the question “Have you suffered psychological stress or acute disease in the past 3 months?” included as part of the Mini Nutritional Assessment Questionnaire [3]. Height and weight were measured by research study staff and body mass index (BMI) was calculated as weight (kg) ÷ height (m^2^). Study participants completed the Vulnerable Elders Survey (VES-13) which is a series of questions to determine risk for health deterioration. The scale ranges from 0 to 10, where a participant with a score of 3 or greater is considered vulnerable and with 10 being most vulnerable [4].

### Laboratory methods

Blood samples were processed, stored, and shipped by each institution’s local Sample Processing Core to Battelle (Columbus, OH). Hemagglutinin inhibition (HAI) testing was performed in duplicate against the influenza vaccine strains in the 2009–2010 Northern Hemisphere influenza vaccine. Although there is debate about the best correlate of protection for influenza [5], seroprotection was defined as an HAI titer of ≥40 since it is the correlate recognized by the United States Food and Drug Administration [6]. Seroconversion was measured at S2 and defined as a four-fold rise in HAI post-influenza vaccination compared to pre-vaccination or ≥40 if S1 was <10. Maintenance of antibody response was measured in participants with an S2 ≥ 40, and defined as an S3 ≥ 40. If duplicate HAI results were discrepant by more than two fold, a third test was performed and the minimum result was recorded.

### Statistical analysis

Multivariable logistic regression models were run for the binary outcomes seroprotection at S2 and S3 and seroconversion at S2. Age, BMI, stress, high risk medical conditions (yes/no), female gender, study site and transformed S1 titer were included in all models. Time in days from S1 to S3 was included in models with S3 related outcomes as a continuous variable. Restricted cubic splines were applied to age and BMI. All raw HAI titers were log transformed using the method outlined by Beyer [7], changing dilution titers to integers with HAI <10 coded as 0, 10 as 1, 20 as 2, 40 as 3 and so on. Interaction terms between age and high risk status, and stress or age and sex were tested and were not included based on non-significant p values of overall interaction terms. Figures were generated by predicting the probability of having a HAI titer ≥40 by logistic regression. All analyses were done using R version 2.12.2.

## Results

A total of 510 participants were enrolled during September and October of 2009 at Vanderbilt University Medical Center (259) and Marshfield Clinic Research Foundation (251). The mean age was 64 years (Interquartile Range [IQR]: 58, 74) with 62% female and 96% white. The participants were very functional with only 9.4% having a VES score of ≥3 and the remaining 91.6% having a score <3. The 494 participants who completed all three visits were similar to the total enrolled; 37% had a high risk medical condition, median BMI was 29 kg/m^2^ (interquartile range [IQR]: 25, 34), and 9% reported having a stressful event in the last 12 months. The median duration between first and third study visit was 257 days (IQR: 250, 263). Data on immunization within the past 3 years were available at Marshfield Clinic; 99% of participants were vaccinated at least once in the previous three years.

Pre-vaccination geometric mean S1 titers for H1N1, H3N2, and B were 7.65, 27.50, and 24.72 respectively. Increasing age was associated with significantly decreased odds of baseline seroprotection for H1N1 (p < 0.01), but not for H3N2 or influenza B. Female sex was associated with decreased odds of seroprotection for influenza B (0.62, 95% CI: 0.41, 0.92), but not for the other strains.

**Figure 1:**
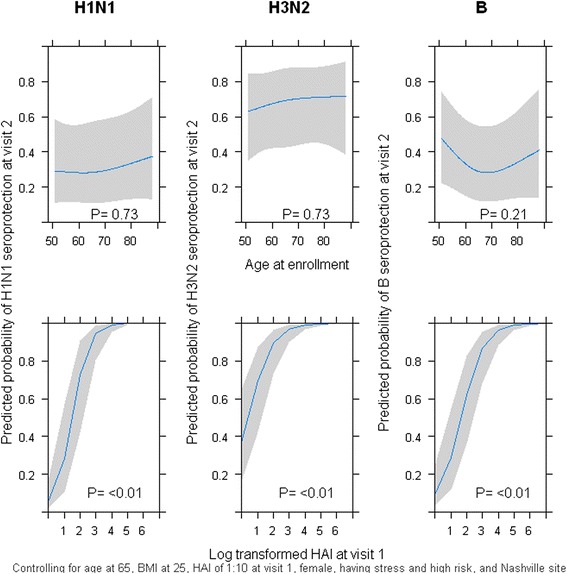
Predicted probability of seroprotection at S2 for each vaccine strain by age at enrollment and pre-vaccination HAI titer. Each graph shows the probability of seroprotection predicted by logistic regression models for H1N1 (first column), H3N2 (second column) and B (third column) by the age at enrollment (first row) or the pre-vaccination HAI titer (second row) 4 weeks post-vaccination.

Post-vaccination responses to H1N1 were the lowest of the three tested strains, with only 12% and 26% of participants achieving seroconversion and seroprotection at S2, respectively. Seroconversion and seroprotection were highest for H3N2 (45% and 82%, respectively; Table 1). Table 2 summarizes the characteristics of participants that achieved seroprotection post-vaccination. There were few prior differences between those who did and did not attain seroprotection at S2 (Table 3). Participants at Vanderbilt were significantly less likely to achieve seroprotection than participants at the Marshfield Clinic for HIN1 and B vaccine strains. Results of multivariable logistic regression models using seroprotection at 28 days post-vaccination as the outcome are shown in Table 3 (binomial variables) and Figure 1 (continuous variables). BMI was not included in the figure since it was not statistically significantly associated with seroprotection for H1N1 or H3N2 (p = 1.0, p = 0.9). Pre-vaccination HAI titer was the only consistent predictor of post-vaccination seroprotection (Figure 1). In sensitivity analyses, excluding all participants with an S1 ≥ 40 prior to vaccination, pre-vaccination HAI titer remained strongly associated with seroprotection at S2 (p < 0.0001) for all three antigens. In this sensitivity analysis, female sex was also associated with seroprotection at S2 for H1N1 (p = 0.0004) and H3N2 (p = 0.04).

**Table 1:** **Proportion of subjects with HAI ≥:1:40 at baseline (S1), 28 days post-vaccination (S2), and after influenza season (S3) and with seroconversion at S2, by influenza strain**

**Seroprotection**	**H1N1**	**H3N2**	**B**
	**% (N)**	**% (N)**	**% (N)**
Baseline (S1)	9% (44)	48% (235)	43% (214)
28 days post-vaccination (S2)	26% (126)	82% (407)	72% (354)
After influenza season (S3)	9% (46)	59% (289)	53% (263)
Seroconversion			
28 days post-vaccination (S2)	12% (60)	45% (222)	28% (138)

**Table 2:** **Enrolled subjects with post-vaccination (S2) titers ≥1:40**

	**H1N1**	**H3N2**	**B**
	**(N=126)**	**(N=407)**	**(N=354)**
	**% (n)**	**% (n)**	**% (n)**
Age (Years)	62 (57,74)	65 (58,75)	66 (58,76)
Sex (Female)	71% (90)	64% (260)	61% (215)
Race (White)	96% (121)	96% (390)	96% (341)
Recent illness or stressor (Yes)	32% (40)	35% (143)	39% (139)
Stress (Yes)	10% (12)	8% (34)	9% (32)
Time from S1 to S3 (days) (mean, IQR)	257 (251,266)	257 (250,264)	257 (251, 264)

**Table 3:** **Baseline characteristics associated with seroprotection post-vaccination (S2) versus not achieving seroprotection**

**Baseline characteristics**	**H1N1**		**H3N2**		**B**	
	Odds ratio*	**p value**	Odds ratio*	**p value**	Odds ratio*	**p value**
Sex (Female vs Male)	3.5	<0.01	2.0	0.02	1.2	0.56
High risk medical condition (Yes vs No)	1.2	0.60	0.8	0.53	1.1	0.74
Recent illness or stressor (Yes vs No)	0.5	0.71	0.8	0.58	0.8	0.60
Site (Vanderbilt vs Marshfield)	0.4	0.01	1.0	0.96	0.5	0.01

*Odds ratios all adjusted for variables in table plus age, BMI, and transformed S1 titer using multivariable logistic regression.

**Figure 2:**
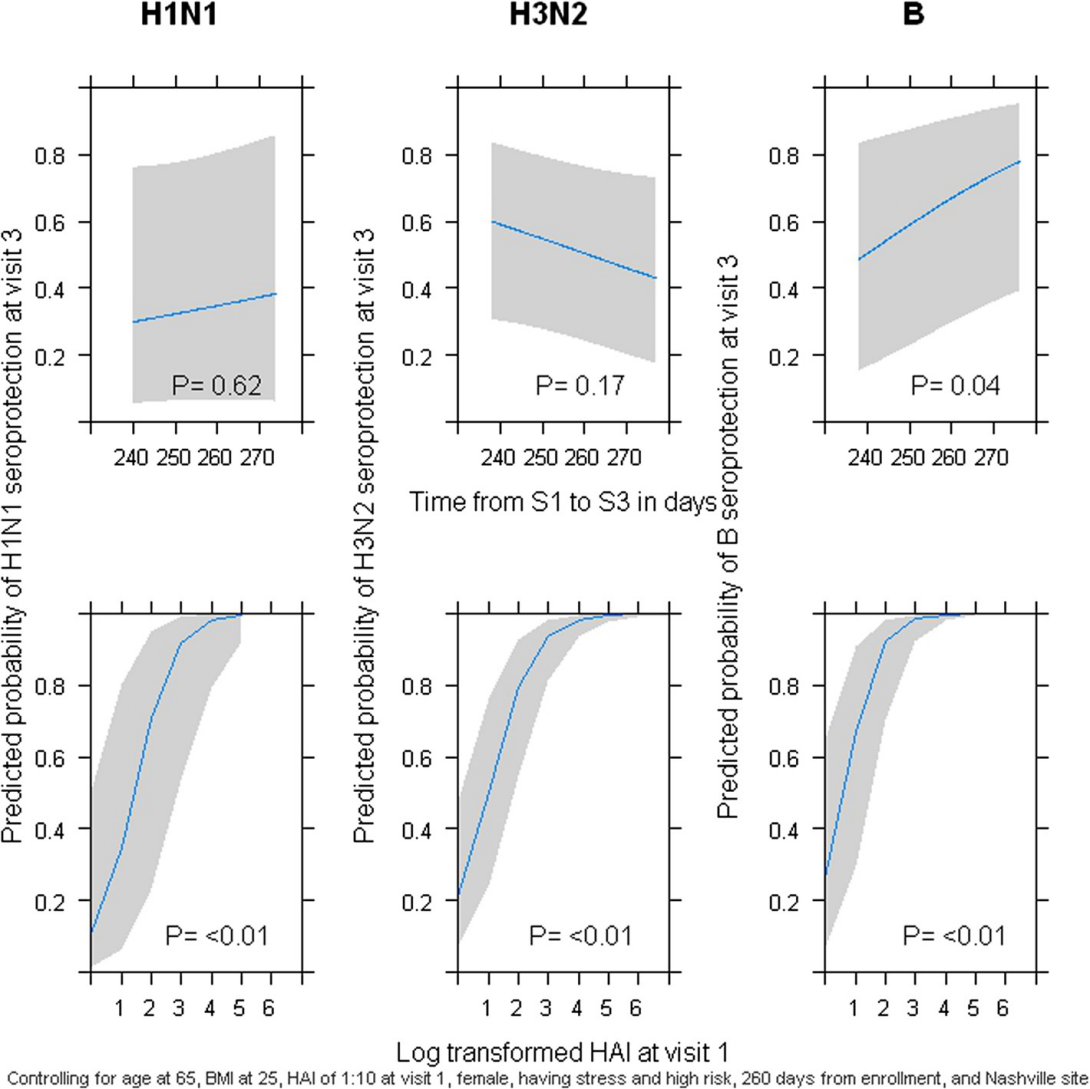
Predicted probability of seroprotection at S3 for each vaccine strain by age at enrollment and pre-vaccination HAI titer. Each graph shows the predicted probability of seroprotection for H1N1 (first column, H3N2 (second column) and B (third column) by the time from vaccination (first row) or the pre-vaccination HAI titer (second row) after influenza season for those with an HAI titer ≥40 after immunization (H1N1, n = 126; H3N2, n = 407; and B: n = 354).

Maintenance of antibody response was assessed in participants who had an S2 HAI titer of ≥40 (Table 4). Maintenance of a protective antibody response was defined by an S3 ≥ 40. Of the participants with an S2 ≥ 40, 36% (46/126), 71% (289/407), and 74% (263/354) maintained an S3 ≥ 40 for H1N1, H3N2, and B influenza vaccine strains, respectively. S1 HAI titer was strongly and consistently associated with maintenance of seroprotection (Figure 2). Higher stress was modestly associated with maintenance of seroprotection for the B strain only (Table 4).

**Table 4:** **Baseline characteristics associated with maintaining versus not maintaining HAI ≥1:40 at S3**

	**H1N1**		**H3N2**		**B**	
	**N=126**		**N=407**		**N=354**	
	Odds ratio*	**p-value**	Odds ratio*	**p-value**	Odds ratio*	**p-value**
Sex (Female)	1.2	0.77	1.1	0.70	0.8	0.46
High risk (Yes)	1.4	0.57	1.7	0.12	0.9	0.70
Recent Illness or stressor (Yes)	3.3	0.16	1.6	0.37	10.6	0.003
Site (Vanderbilt vs Marshfield)	2.3	0.11	1.6	0.14	1.4	0.30

*Odds ratios all adjusted for variables in table plus age, BMI, days between S3 and S1 and transformed S1titer using multivariable logistic regression.

## Discussion

In our cohort of adults ≥50 years of age, the initial antibody response to influenza vaccination and maintenance of seroprotective levels of antibody response throughout the influenza season were strongly associated with pre-vaccination antibody levels for all three vaccine strains.

A number of prior studies have assessed the duration of antibody response in older adults, but few have determined factors associated with maintenance of protection [8-17]. Prior studies have been inconsistent about duration of protection with results ranging from 15% maintaining seroprotection at 5 months [8] to 88% maintaining seroprotection at 12 months [16]. Some studies showing prolonged maintenance may have been complicated by interval natural infection. Our results are unlikely to have been confounded by natural influenza infection since the only influenza virus strain circulating was the pandemic strain, not yet included in the seasonal trivalent influenza vaccine.

A study in Korean adults ≥65 years of age was one of the few prior studies to assess factors associated with maintenance of antibody levels. Similar to our results, maintenance of antibody response was associated with pre-vaccination HAI titer ≥40. In this population, maintenance was also associated with less advanced age [18]. This latter difference in findings could be due to the older age of participants (mean age 71.7 ± 4.5) in the Korean study compared to the mean age of 64 ± 10.2 years of our participants.

Interestingly, we also found differences between the two study sites. Participants at Vanderbilt were less likely to attain seroprotective levels of antibody to influenza A H1N1 and influenza B, controlling for other factors. Age criteria for enrollment differed at the two sites, but these differences in attaining protective titers persisted after controlling for age. Participants at Marshfield Clinic were clinic patients recruited from those vaccinated the prior year; whereas participants at Vanderbilt were volunteers. It is possible that differences in vaccination or prior influenza disease not reflected in baseline S1 were responsible for differences observed. Differences were unlikely due to vaccination in the prior year since >97% had received influenza vaccine in the year prior. Other explanations are differences in the specific vaccines administered or methods of administration, chance, or some other unmeasured factor.

Both sites had very low seroconversion rates to H1N1 and overall low seroprotection. Goodwin et al. summarized results from 31 studies of influenza vaccine responses in elderly adults 1986—2002, and reported an average seroconversion rate of 42% and a 69% seroprotection rate to H1N1 viruses [19]. However, there is considerable year to year variability, and a seroprotection rate of 11% was reported in a study done during the 1993–1994 season among adults ≥65 years of age when the H1N1 vaccine strain was A/Texas/36/91 [20].

This study was limited by several factors. Foremost, this is a single study year with a single seasonal influenza vaccine. Vaccine strains can change each year. In the 2009–2010 Northern Hemisphere influenza vaccine, both the H1 and the H3N2 components had been used during the prior years, but the B component for the 2009 vaccine was new. The repetitive use of an antigen may make pre-vaccination results more important than for a novel antigen. However even for the B antigen, pre-vaccination response remained the most prominent factor associated with vaccine response. Because most participants had been vaccinated within the past year, it was not possible to determine the effect of prior immunization. The results of this study may not be generalizable to very old adults, since the mean age was only 64 years of age and participants were generally healthy with 91.6% classified as not vulnerable. Lastly, an antibody titer of ≥40 was chosen as the definition of seroprotection because this is the level of antibody used for influenza vaccine licensure [6]. It is unclear if this is a reliable predictor of protection in older adults. Even in younger adults, titers ≥40 have been seen in cases of influenza vaccine failure [5].

The study clearly demonstrated that antibody response to trivalent inactivated influenza vaccine, and maintenance of this response, are associated with pre-vaccination antibody titers. Hence, older adults with low pre-vaccination HAI antibody titers are less likely to respond to influenza vaccination. It is unclear if these older adults are at higher risk for influenza and complications to influenza or if they may be vaccine failures. Future research will need to determine if this places these adults at higher risk, and whether specific types of vaccines will result in a more robust immune response and a greater likelihood of protection in these older adults.

## Conclusions

In summary, pre-vaccination antibody titer was the only study variable consistently and positively associated with both serologic response to vaccination and maintenance of response in older adults. Antibody responses were lowest for the H1N1 vaccine strain despite less severe disease in older adults due to H1N1 compared to other strains.

## Abbreviations

BMI: Body mass index
CDC: Centers for Disease Control and Prevention
HAI: Hemagglutinin inhibition
IQR: Interquartile ratio
